# Isothiocyanate-rich moringa seed extract reduces skin inflammation in mouse ear edema model

**DOI:** 10.1016/j.phyplu.2023.100479

**Published:** 2023-08-16

**Authors:** Khea Wolff, Keyaara Robinson, Nanjoo Suh, Bozena Michniak-Kohn, Michael Goedken, Marianne Polunas, Ilya Raskin

**Affiliations:** a Department of Plant Biology, School of Environmental and Biological Sciences, Rutgers, The State University of New Jersey, New Brunswick, New Jersey, United States of America; b Ernest Mario School of Pharmacy, Rutgers, The State University of New Jersey, New Brunswick, New Jersey, United States of America; c Rutgers Office for Research, Rutgers, The State University of New Jersey, New Brunswick, New Jersey, United States of America

**Keywords:** Moringa seed extract, Moringa isothiocyanate-1, Ear edema, Inflammation, Cytokines, Chemokines

## Abstract

**Background:**

Moringa (*Moringa oleifera* Lam.) seed extract (MSE) and its primary bioactive compound, moringa isothiocyanate-1(MIC-1), mitigate inflammation, oxidative stress, diabetes, and cancer in the *in vivo* rodent models following oral application.

**Purpose:**

To investigate the topical anti-inflammatory activity of MSE and purified MIC-1 in a TPA-induced mouse ear edema model.

**Study Design:**

The present study elucidates the topical anti-inflammatory effects and mechanisms of action of MSE, containing 38% of MIC-1 and purified MIC-1 using a mouse ear edema model utilizing 12-O-tetradecanoylphorbol-13-acetate (TPA), as the pro-inflammatory agent.

**Methods:**

A time-dependent and dose-dependent response was determined by pretreating CD-1 mice with various doses of MSE and MIC-1, positive control, dexamethasone, or vehicle control, followed by TPA, and the subsequent difference in ear thickness was measured using digital Vernier calipers. The effective doses of MSE and MIC-1were then selected to evaluate the change in weight of the ears using 6 mm biopsy punches and the results were confirmed by microscopy. Inflammatory markers were quantified with Luminex multiplex immunoassay.

**Results:**

MSE and MIC-1 were effective in a dose-dependent manner in a TPA-induced ear edema model, causing a reduction in ear thickness and a 48% and 49% decrease in ear punch weight, respectively. MSE and MIC-1 also caused a reduction in the levels of cytokine and chemokines, interleukin 6 (IL-6), monocyte chemoattractant protein-1 (MCP-1), and keratinocyte chemoattractant (KC) in the ear tissue. MSE and MIC-1 reduced IL-6 expression by 84% and 78%, MCP1 by 74% and 73%, and KC by 56% and 43%, respectively. Additionally, the anti-inflammatory effect of MSE and MIC-1 was confirmed by hematoxylin and eosin (H&E) staining, used to assess the thickness of the ear swelling. MSE significantly reduced the thickness of the ears by 20% compared to TPA.

**Conclusion:**

These results reveal the topical anti-inflammatory properties of MSE, and MIC-1 likely transmitted via the nuclear factor erythroid 2-related factor 2 (Nrf2) and nuclear factor-kappa B (NF-κB) pathways as mentioned in previous studies. This work also suggests therapeutic uses of MSE and/or MIC-1 for skin inflammation.

## Introduction

In addition to its barrier function, inflammation is the skin’s natural defense mechanism against a foreign substance (Xu et al., 2017). There is an extensive relationship among epithelial, stromal, and immune cells that regulate the skin’s immune response to ensure an effective defense and maintain homeostasis (Pasparakis et al., 2014). When an offending agent such as bacteria, chemicals, or injury is present, the early inflammatory response attracts neutrophils and macrophages directly to the site. This elicits two responses, innate or adaptive, both of which involve immune cells that target the site of inflammation (Braun et al., 2002). The severity of topical inflammatory conditions can range from acute to chronic and include disorders such as atopic dermatitis, psoriasis, and hidradenitis suppurativa (Farzanfar et al., 2018). All of these conditions involve the recruitment and activation of specific immune cells that are regulated by transcription factors such as Activator Protein 1 (AP-1), Nuclear Factor-кB (NF-кB), Nuclear Factor of Activated T-cells (NFATs), Signal Transducer and Activator of Transcription (STATs) proteins, and production of numerous cytokines and chemokines (Uluçkan et al., 2015, Nutten, 2015).

While oral non-steroidal anti-inflammatory drugs (NSAIDs) are typically prescribed for the treatment of inflammatory conditions, they are responsible for about one-quarter of all adverse drug reactions reports that are made (Heyneman et al., 2000). Given the burden that inflammatory skin conditions have on older adults and persons who cannot be prescribed oral drug products for extended periods, the use of topical NSAIDs or corticosteroids has been increasingly preferred by patients and prescribed by healthcare providers (Makris et al., 2010). The introduction and use of topical corticosteroids is the most significant contribution to the treatment of dermatological disorders. However, patients may develop side effects as a result of prolonged use of topical treatments for inflammation. For example, cutaneous atrophy, steroid rosacea, perioral dermatitis, the development of telangiectasia (widened blood vessels), and skin infections are a few of the side effects that have been observed (Horn et al., 2010).

Commonly known as moringa, *Moringa Oleifera* Lam., is one such plant which has been shown to be effective against inflammation. Moringa is traditionally grown and cultivated in tropical and subtropical climates and has been used for many years as food and medicine. The Moringacea and Brassicaceae families contain a group of compounds called glucosinolates which are converted to isothiocyanates by the enzyme myrosinase. However, moringa’s isothiocyanates have unique properties that make them particularly suitable for clinical applications. Firstly, moringa isothiocyanates are stable, solid compounds, due to the presence of a rhamnose group on the benzyl ring, which is not present in the Brassicaceae isothiocyanates (Waterman et al., 2014). Moreover, moringa isothiocyanate-1 (MIC-1) can be produced in large quantities from moringa seeds and easily purified (Jaja-Chimedza et al., 2017). Additionally, MIC-1 is highly bioavailable and remains largely unmodified during uptake, unlike Brassicaceae isothiocyanates (Wolff et al., 2020).

MIC-1 and moringa seed extract (MSE) (Jaja-Chimedza et al., 2017) enriched in MIC-1 to 30–40% w/w were highly effective in activating genes responsible for oxidative stress protection (antioxidant genes) that are regulated by nuclear factor erythroid 2-related factor 2 (Nrf2) transcription factor (Boyunegmez Tumer et al., 2015, Jaja-Chimedza et al., 2017). Nrf2 activation was suggested earlier as a cellular target for Brassicaceae isothiocyanates, such as sulforaphane (Kubo et al., 2017). We also observed that MIC-1 was an effective inducer of anti-inflammatory genes regulated via the NF-κB pathways (Kim et al., 2017). Our recent immuno-cytochemical studies directly demonstrated that MIC-1 induces Nrf2 translocation and inhibits NF-κB translocation into the nuclei of the lipopolysaccharide (LPS)-induced murine macrophages (Sailaja et al., 2021). Moringa isothiocyanates also showed apoptotic activity in human prostate cancer cells (Abd Karim et al., 2023).

Earlier mouse *in vivo* studies showed that orally administered moringa isothiocyanate-enriched extract or MIC-1, mitigated obesity-related metabolic dysfunction (Sailaja et al., 2021, Jaja-Chimedza et al., 2018, Kim et al., 2018, Kim et al., 2017); attenuated ulcerative colitis symptoms (Kim et al., 2017); and reduced LPS-induced sepsis / acute inflammation (Sailaja et al., 2022), while showing relatively low toxicity in a 14-day repeated- oral dose study in rats (Kim et al., 2018). We also observed induction of antioxidant and anti-inflammatory genes in mouse liver, kidney, spleen (Sailaja et al., 2021), colon (Kim et al., 2017) and muscles (Sailaja et al., 2022) in the mentioned *in vivo* studies. However, differently prepared lipid-rich moringa seed extract showed some kidney toxicity (Attah et al., 2022).

This study evaluates the ability of an isothiocyanate-rich MSE and its main active, MIC-1, to reduce inflammation in a 12-O-tetradecanoylphorbol 13-acetate (TPA)-induced mouse ear edema model. This study is significant because this is the first report of topical anti-inflammatory effect of moringa isothiocyanates, that suggests their effectiveness for skin applications. We found that both MSE and MIC-1 suppressed skin inflammation in a dose-dependent manner, reduced the thickness of the skin in the ear model, and decreased cytokine and chemokine, MCP-1, KC and IL-6 infiltration. These are important markers involved in the inflammatory process, specifically the NF-кB pathway. We suggest that MSE and MIC-1 may be further studied and possibly developed as natural, topical anti-inflammatory agents.

## Materials and methods

### Preparation of MSE and MIC-1

The moringa seed extract (MSE) containing 38% of MIC-1 w/w was prepared from seeds obtained from The Jamaica Moringa Farmer’s Association (Kingston, Jamaica), and was prepared as previously described (Jaja-Chimedza et al., 2017). Briefly, moringa seeds were weighed, ground in a blender, and incubated on a shaker kept at 37 °C for 2 h in water at a ratio of 1 g seed powder: 3 ml water. Thereafter, ethanol was added to the mixture in a ratio of 1 ml water: 4 ml ethanol, and the slurry filtered and dried using a rotary evaporator and a freeze drier. The MSE was then stored at −20 °C. MIC-1 content in MSE was quantified by LCMS as described (Wolff et al., 2020). MIC-1 was isolated and purified by resuspending 2 g of the freeze-dried MSE in a 10 ml solvent system comprising a mixture of hexane, ethyl acetate, methanol, and water in a 4:6:4:6 ratio (Friesen and Pauli, 2015). The resuspension was vortexed, filtered, sonicated for 10 minutes, and subjected to Fast Centrifugal Partition Chromatography (liquid-liquid chromatography) in FCPC^®^1000 Kromaton v1.0. The wavelength on the UV detector was set to 229 nm. The peak at about 100 min was collected, and solvents then removed via rotary evaporation and freeze drying. Purified MIC-1 (at least 90% purity) is a white, crystalline powder and was stored at −20 °C for later use. For animal experiments, MSE and MIC-1 were prepared in a 10% ethanol solution.

### Mice

Animal studies were approved by the Institutional Animal Care and Use Committee (IACUC) of Rutgers University (protocol # ID999900474), following ethical guidelines. Male CD-1 mice, 6–8 weeks old, were purchased from Charles River Laboratories (Malvern, PA) and acclimated for one week under controlled temperature (22 ± 2°C), humidity (40–60%), and in a light/dark cycle for 12 h. The experimental and control mice were co-housed, 4 per cage, and allowed access to food and water *ad libitum*.

### TPA-induced mouse ear edema model

Mice were randomly assigned to five treatment groups and anesthetized under 2–5% isoflurane for 10 minutes for the ear edema experiments. TPA was prepared at a concentration of 0.1 μg/μl (2 μg/ear) and dexamethasone was prepared at a concentration of 2.5 μg/μl (0.05 mg/ear) in acetone. MSE and MIC-1 were both prepared in 10% ethanol, with the MSE dosed at 6.4 (0.128 mg/ear), 12.8 (0.256 mg/ear), 50 (1 mg/ear), 100 (2 mg/ear) μg/μl in 20 μl. MIC-1 doses included 2.5, 5, 20, 40 μg/μl or 0.05, 0.1, 0.4, 0.8 mg/ear in 20 μl.

‘Untreated control’ animals received no ear treatment or moringa preparations. ‘TPA’ animals received only TPA on the right ear, 2 μg/ear, and 20 μl of the vehicle control, acetone, on the left. The ‘TPA + dexamethasone’ group, or positive control animals, received 0.05 mg in 20 μl dexamethasone in acetone on the right ear followed by TPA, with the left control ear receiving 20 μl of acetone. The ‘TPA + MSE’ group received the dose of MSE (dependent on the experiment) and then TPA, with the left ear receiving 20 μl of both the vehicle controls, 10 % ethanol followed by acetone, the vehicles for MSE and TPA respectively. Similarly, for the ‘TPA + MIC-1’ group, receiving the specific dose of MIC-1 on the right ear, followed by TPA, with the left ear receiving 10 % ethanol and then acetone. Consistent for all treatments was the administration of TPA 20 min posttreatment application.

At each time point, 2, 4, and 8 h post-TPA treatment, the ear thickness was measured using digital Vernier calipers. A sterile 6 mm biopsy punch (VWR) was used to collect the biopsy samples which were then used for cytokine and chemokine evaluation with LUMINEX as reported (Wu et al., 2020).

### Ear tissue and serum collection

The animals were euthanized via CO_2_ inhalation and then cardiac puncture to prepare the samples for cytokine and chemokine analysis. The blood collected from the cardiac puncture was allowed to clot for 30 min at room temperature before centrifugation to collect the serum. After cardiac puncture, 6 mm biopsy punches of the ear tissue were collected and immediately placed into tubes with homogenizing beads and extracted using 10 ml of Tissue Extraction Reagent, a Tris-based lysis buffer for total protein extraction, supplemented with proteinase inhibitor, for every 1 g of tissue. For serum collection, the blood was centrifuged at 1500 × *g* for 10 min and then placed in Eppendorf tubes on ice. The ear tissue samples were homogenized, centrifuged, and the supernatant collected. The amount of protein was quantified using a standard protein assay kit. The ear tissue samples were prepared at 1 μg/μl of protein and along with the serum samples, analyzed at Cancer Institute of New Jersey core facility.

### LUMINEX analysis

The Luminex assay was performed following the vendors’ protocol at the Rutgers Cancer Institute of New Jersey Immune Monitoring Shared Resource. Briefly, cytokines and chemokines were measured using the 10-plex Millipore Milliplex Catalog ID. MCYTOMAG-70K-10 MouseCytokine MAGNETIC Kit which contains; interleukin-1α (IL-1α), interleukin-6 (IL-6), tumor necrosis factor-α (TNF-α), interleukin-1B (IL-1B), interleukin-10 (IL-10), monocyte chemoattractant protein-1 (MCP-1), keratinocyte chemoattractant (KC), interferon gamma (IFNγ), interleukin-12p40 (IL-12p40), interleukin-12p70 (IL-12p70). Ear protein samples and serum samples were analyzed on a 96-well plate. Using a Luminex 200 system with Luminex xPONENT software (Luminex Corp.; Austin, TX, USA) using a 5PL curve. For a given sample and analyte, concentration values were discarded if readings were <30 beads. Analytes falling above or below these values were inputted to the lower or upper limit of quantification, respectively.

### H&E staining

Two different subsets of animals were used (n = 14 each) to collect ear biopsy samples and whole ears for H&E staining. Staining and analysis was performed by the Research Pathology Services in the Office for Research (Rutgers, New Jersey, USA). Whole ear samples were collected 7 h posttreatment and fixed in 10% neutral-buffered formalin solution at room temperature. The H&E staining from 4–6 biological replicates per treatment group was performed blinded. During the preparation and evaluation of the slides, the pathologist was also blinded to the treatments performed for each sample. The Aperio ImageScope software was used to analyze the thickness of the slides at three random locations in the images of each of the treatments.

### Statistical analyses

Statistical analysis was performed using GraphPad Prism Version 9. Comparisons between treatment groups were performed using one-way and two-way ANOVA, followed by Tukey’s multiple comparison test. A *p* value < 0.05 was considered statistically significant.

## Results

**Fig. 1.**
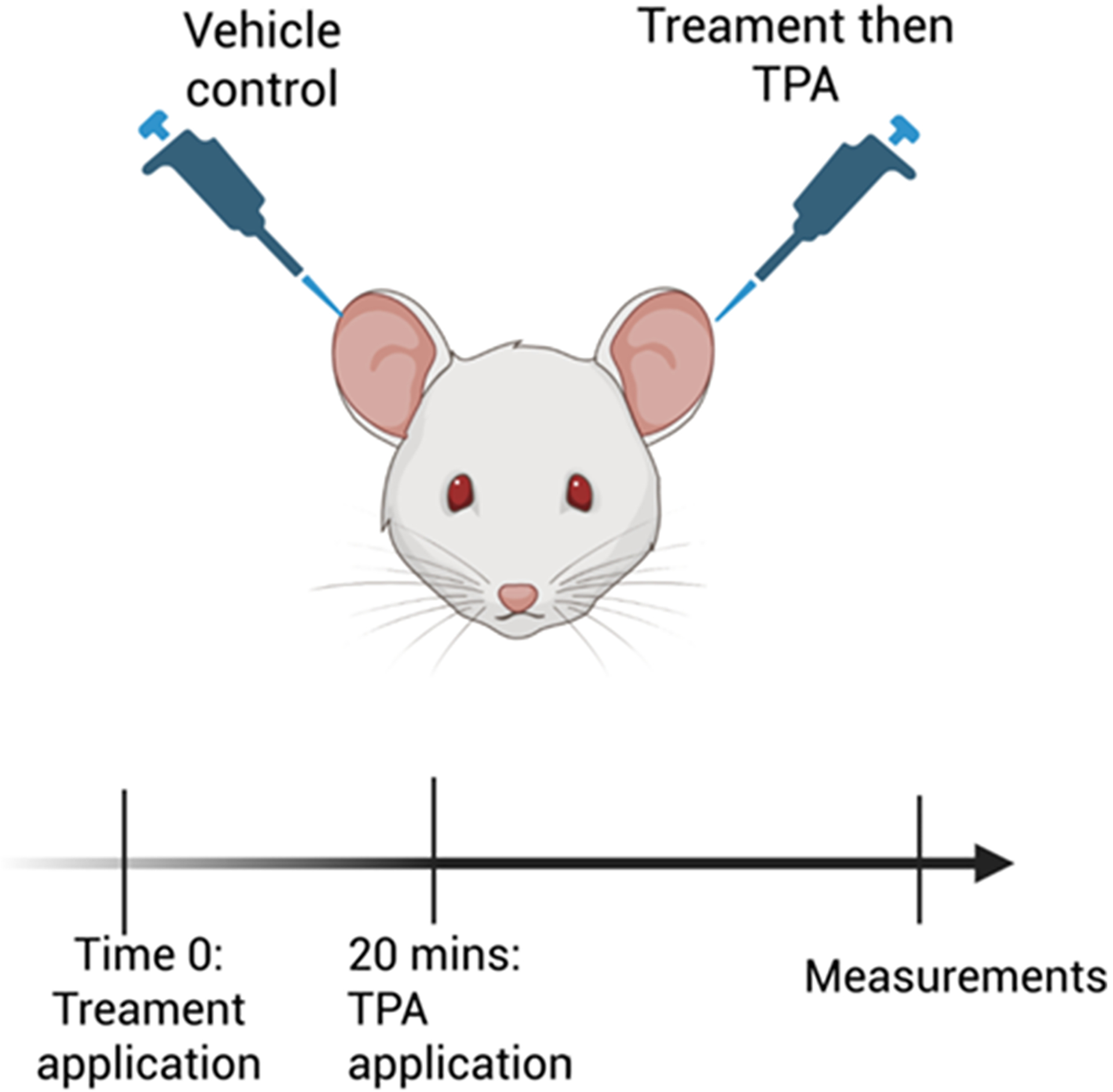
Diagram of the experiment design for the TPA-induced ear edema model. The right ear of the animals received the experimental treatment (dexamethasone at 0.05 mg/ear, MSE at 0.128, 0.256, 1, or 2 mg/ear, MIC-1 at 0.05 mg, 0.1 mg, 0.4 mg and 0.8 mg/ear) applied 20 min before TPA (2ug/ear), while the left ear received the vehicle control (20 μl of 10% ethanol and/or acetone) at the time of the TPA treatment. Untreated control animals received no treatment, while TPA animals received TPA on the right ear and acetone on the left. For the ear thickness, the measurements were taken at 2- and 4-h post-TPA application. For the ear biopsy experiments, measurements were taken 4 h post-TPA application. For the H&E staining, ears were collected at 7 h post-TPA treatment.

**Fig. 2.**
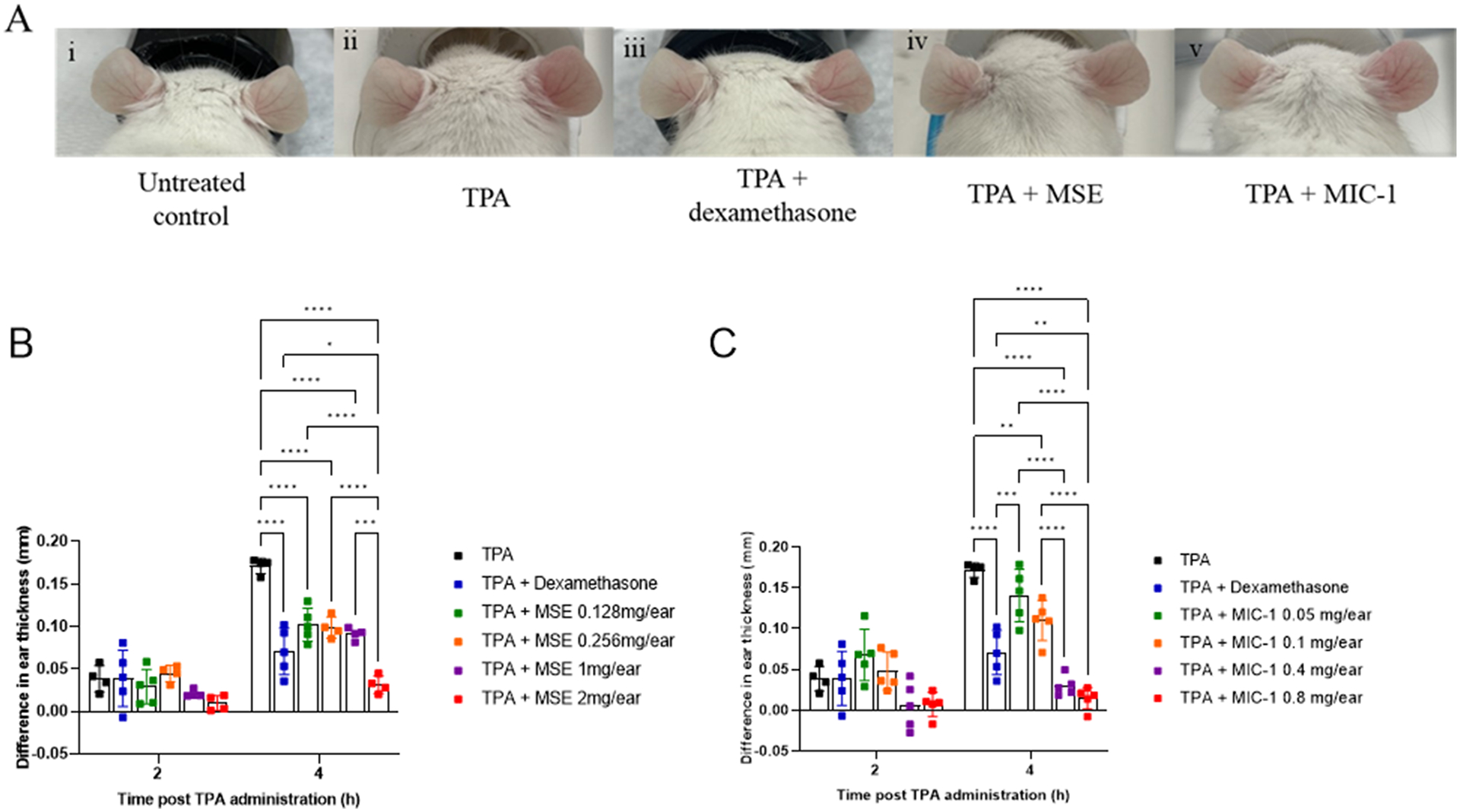
Images of mouse ears subjected to experimental treatments (A). Effects of MSE-1 (B) and MIC-1 (C) on the TPA-induced ear edema of the right ear, measured as reduction of the ear thickness compared to the untreated 20 μl of 10% ethanol and/or acetone left ear. Photos represent each of the treatment groups, TPA (2 mg/ear), TPA + dexamethasone (0.5 mg/ear), TPA + MSE (2 mg/ear) and TPA + MIC-1 (0.8 mg/ear). TPA was administered 20 min posttreatment. The photos were taken 4 h post-TPA administration. Data (mm) were expressed as difference in thickness between treated (right) and untreated (left) ears. Data are represented as mean ± SD of 4–5 animals. For the baseline, the ear thickness was measured at 0 h. Statistical significance was determined by two-way ANOVA followed by Tukey’s post-hoc test; *p < 0.05, **p < 0.005, ***p < 0.0005, ****p < 0.0001. No significance was observed at 2 h.

To investigate the dose and time effects of MSE and MIC-1 on the TPA-induced ear inflammation, the ear thickness was measured at 0 h for the baseline, then at 2 and 4 h post-TPA application as per experimental diagram (Fig. 1). The animals were pre-treated on their right ears with either the vehicle, 0.5 mg/ear dexamethasone, varying concentrations of MSE, 0.128 mg, 0.256 mg, 1 mg, and 2 mg/ear in 10% ethanol or the equivalent dose of MIC-1, 0.05 mg, 0.1 mg, 0.4 mg, and 0.8 mg/ear also in 10% ethanol. The left ears received 10% ethanol and/or acetone as the vehicle controls. To quantify the treatment’s effect, the difference between the thickness of the right ear and the left control ear was calculated. Data indicated that 4 h is the least amount of time, post-TPA administration, to observe a statistically significant response in edema (Figs. 2B–C). Fig. 2A shows images of the changes in the ear morphology at the 4 h time point for each treatment. Untreated ears (i) are thin and white, thinner towards the tips with visible capillaries. The right ear of the TPA treatment (ii) is thicker and redder in appearance than the left control ear of the animal and compared to the right ears of the other treatment groups. The animals that received the TPA + dexamethasone (iii), TPA + MSE (iv) and TPA + MIC-1(v) showed a reduction in the TPA-induced swelling and redness of the right (treated) ear.

Four hours after treatment, there was a statistically significant decrease in the ear thickness in the TPA + MSE group at all doses as compared to the TPA group (Fig. 2B). TPA alone increased the thickness of the right ear by 0.171 ± 0.01 mm. The lowest concentration of MSE, 0.128 mg/ear, reduced thickness deferential to 0.102 ± 0.02 mm, or a 40% decrease compared to TPA alone. MSE at 0.256 mg/ear reduced thickness deferential to 0.099 ± 0.01 mm, or a 42% decrease, MSE at 1mg/ear resulted in 0.091 ± 0.01 mm thickness differential or a 47% decrease and MSE at 2 mg/ear produced 0.032 ± 0.01 mm differential, an 81% decrease compared to TPA treatment. The positive control, dexamethasone, reduced the swelling thickness by 0.075 ± 0.012 mm or 58%. There was no statistical difference observed at 2 h for any of the treatments. A similar trend 4 h posttreatment was observed in animals treated with TPA and MIC-1 (normalized for a dose provided by the MSE), with a decrease in ear thickness of 0.141 ± 0.03 mm or 18% for MIC-1 at 0.05 mg/ear, 0.110 ± 0.03 mm or 36% for MIC-1 at 0.1 mg/ear, 0.030 ± 0.01 mm or 82% for MIC-1 at 0.4 mg/ear and 0.014 ± 0.01 mm or 92% for MIC-1 at 0.8 mg/ear (Fig. 2C). Again, there was no statistically significant reduction in thickness at 2 h. MSE at 2 mg/ear and MIC-1 at 0.4 mg/ear and 0.8 mg/ear outperformed the positive control dexamethasone. Therefore, MSE at 2 mg/ear and MIC-1 at 0.8 mg/ear were selected for further experiments.

### MSE and MIC-1 reduce ear biopsy punch weights

**Fig. 3.**
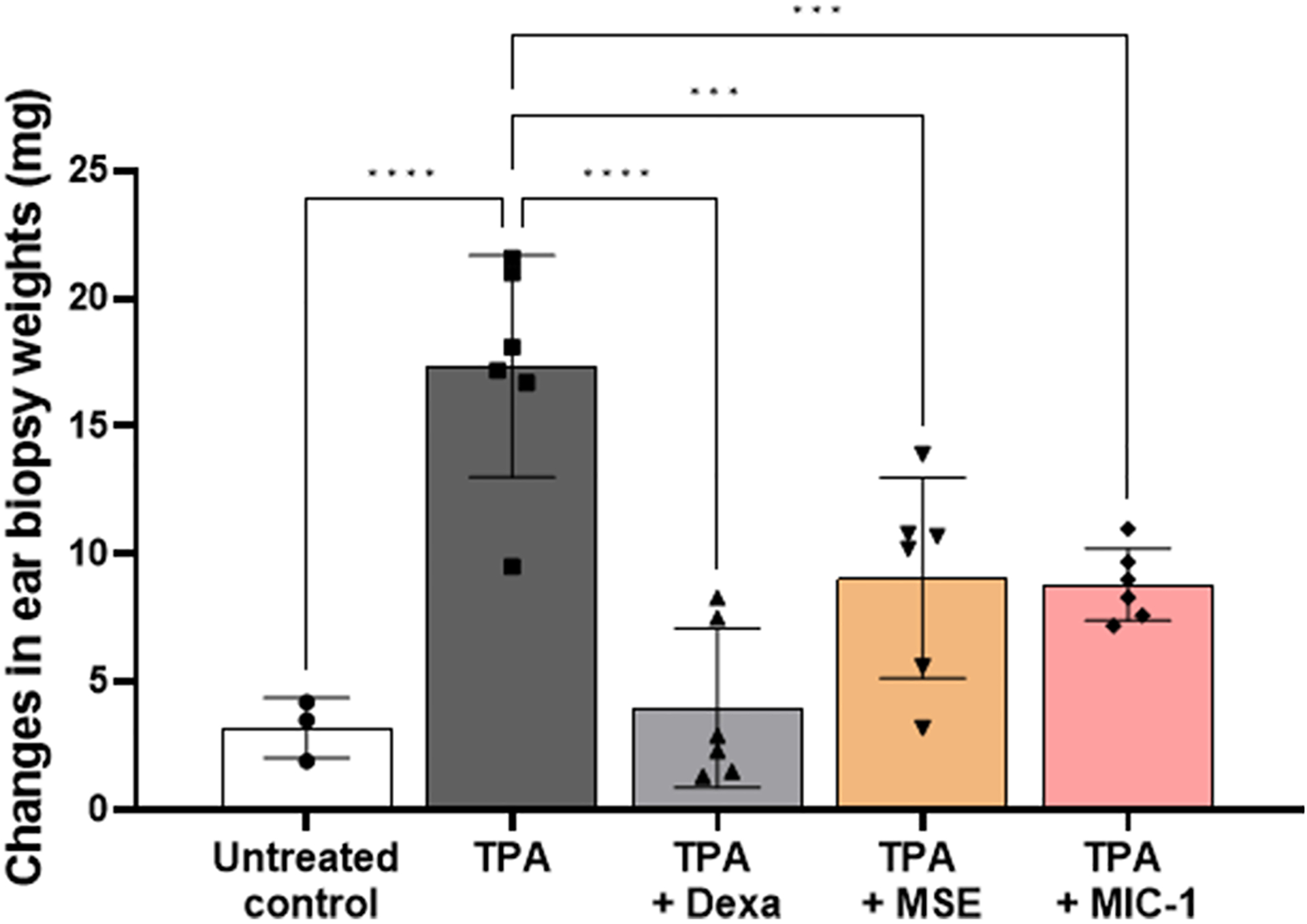
Effects of MSE and MIC-1 on the attenuation of the TPA-induced ear edema measured as differences in weights (mg) of 6 mm ear punch biopsies. Treatment groups included untreated control, TPA (2 mg/ear), TPA + dexamethasone (0.5 mg/ear), TPA + MSE (2 mg/ear) and TPA + MIC-1 (0.8 mg/ear). TPA was administered 20 min posttreatment. Biopsy punches were collected 4 h post-TPA administration. Data are expressed as a difference between the weights (mg) of the biopsy punches from the treated (right) and untreated (left) ears. Data are represented as mean ± SD of 4–5 animals. Statistical significance was determined by one-way ANOVA followed by Tukey’s post-hoc test; **p < 0.005, ****p < 0.0001.

Six mm ear biopsy samples were collected using sterile biopsy punches from the control (left) and treated (right) ears. The biopsy punches were weighed, and the results calculated as the difference in ear punch weight in mg compared to the controlled left ear (Fig. 3). Untreated control ears showed a change of 3.2 ± 1.2 mg in the punch weight deferential, suggesting that the left and right ear of the mice are not identical in size or thickness. The weight differential increased to 17.4 ± 4.3 mg, compared to the control (left ear) punches, for the animals receiving TPA alone, a 443% or 4-fold increase. Dexamethasone limited the TPA-induced weight gain to a 25% increase with a 4.0 ± 3.1 mg change, MSE at 2 mg/ear caused a 184% increase or 2-fold increase in TPA-induced weight gain of the biopsy punches or 9.1 ± 3.9 mg and MIC-1 at 0.8 mg/ear, resulted in a 175% increase with a change of 8.8. ± 1.4 mg.

### MSE and MIC-1 selectively decrease levels of MCP-1, KC, and IL-6 in mouse ear tissue

**Fig. 4.**
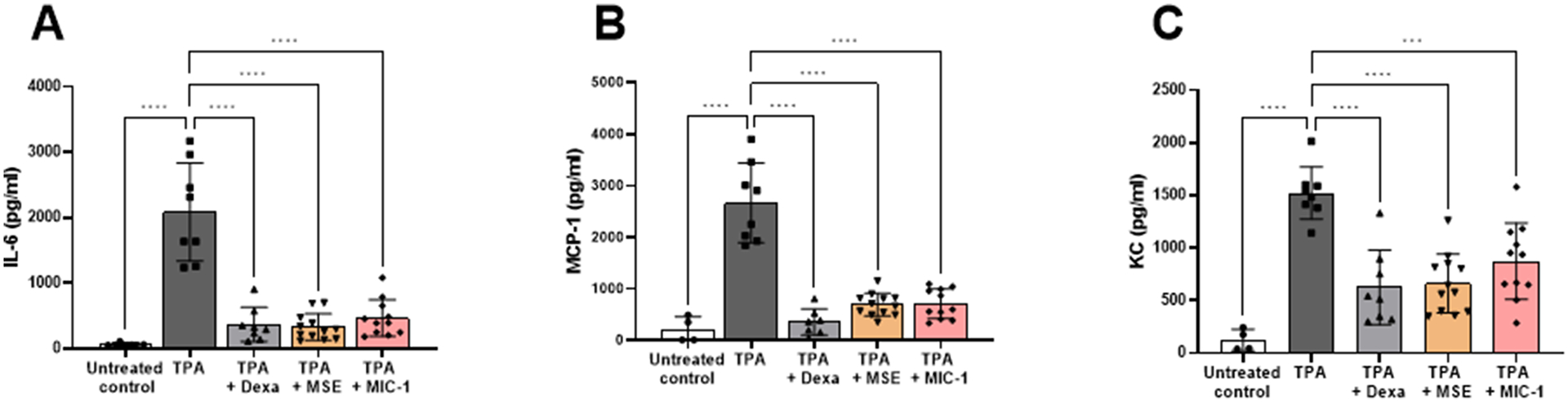
MSE and MIC-1 suppressed the expression of (**A**) IL-6 and (**B**) MCP-1 and (**C**) KC in TPA inflamed ear tissue. Ear biopsy samples, 6 mm, were collected 4 h post-TPA administration. Data shown as mean ± SD and are representative of two independent experiments of 4–8 mouse ear samples. Statistical significance was determined by two-way ANOVA followed by Tukey’s post-hoc test; ***p < 0.0005, ****p < 0.0001.

To evaluate the effects of MSE and MIC-1 on the production of the inflammatory markers in the ear tissue, LUMINEX assay was performed on 6 mm ear biopsy punch samples, 4 h post-TPA application. Similarly, to the effect of positive control, dexamethasone, MIC-1 at 0.8 mg/ear and MSE at 2 mg/ear significantly reduced the levels of chemokines MCP-1 and KC along with cytokine IL-6 in the lysate of the ear tissue (Fig. 4). Control left ears expressed IL-6 at 69 ± 26.2 pg/ml. In contrast, the TPA-treated right ear boosted IL-6 production to 2085 ± 751.1 pg/ml, a 2922%- or 29-fold increase (Fig. 4A). MSE and MIC-1 lowered the TPA-induced levels of IL-6 by 84.2% and 77.6% (330 ± 200.8 pg/ml and 467 ± 278.2 pg/ml, respectively). Dexamethasone brought the TPA-induced IL-6 levels down by 82% or to 369 ± 260.4 pg/ml.

Chemokine MCP-1 showed a similar trend. Control left ear expressed MCP-1 at 219 ± 245.2 pg/ml, while the TPA-treated right ear boosted MCP-1 production to 2669 ± 767.8 pg/ml, a 1119% or 11-fold increase (Fig. 4B). MSE and MIC-1 lowered the TPA-induced levels of MCP-1 in the right ear by 74% and 73%, to 698 ± 219.7 pg/ml and 713 ± 286.7 pg/ml, respectively (Fig. 4B). Dexamethasone reduced MCP-1 expression by 86% to 362 ± 256.4 pg/ml.

A chemokine KC in (Fig. 4C) was affected similarly to the previous two markers. Control, left ear expressed 126 ± 101.5 pg/ml of KC. MSE and MIC-1 significantly reduced TPA-induced KC expression in the right ear by 56% and 43%, respectively to 662 ± 278.6 pg/ml and 874 ± 364.2 pg/ml when compared to TPA-induced expression at 1522 ± 247.8 pg/ml. Dexamethasone performed similarly, reducing the expression by 59% at 626 ± 355.4 pg/ml. Effects of MSE and MIC-1 on IFN-g, IL-1α, IL-1B, IL-10, IL-12p40, IL-12p70 and TNF-α were also measured. These inflammatory markers did not show statistically significant changes in response to the experimental treatments (data not shown).

### MSE and MIC-1 selectively reduce IL-6 and KC level in serum

**Fig. 5.**
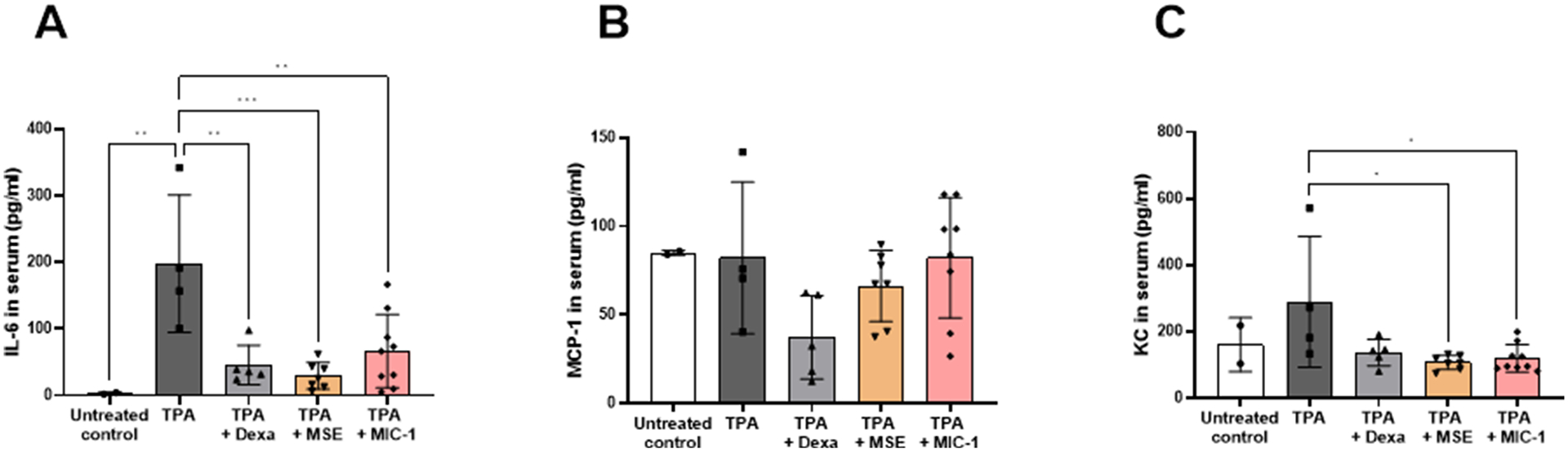
Effects of MSE and MIC-1 on (**A**) IL-6 and (**B**) MCP-1 and (**C**) KC in serum. Serum was collected 4 h post-TPA administration. Data shown as mean ± SD and are representative of two independent experiments of 4–24 ear samples. Statistical significance was determined by two-way ANOVA followed by Tukey’s post-hoc test; *p < 0.05, **p < 0.005, ***p < 0.0005. No significance was observed for chemokine MCP-1.

To determine whether topical treatment with MSE and MIC-1 exhibit a systemic response beyond the ear tissue, blood serum from the experimental animals was collected and analyzed. The trend of IL-6 in serum was similar to IL-6 in the tissue lysate with the difference being the amount of the cytokine expressed. There was a 90% decrease in the amount of IL-6 in the serum than in the ear tissue lysate with the TPA-induced levels in the lysate at 2085 ± 751.1 pg/ml and the TPA-induced levels in the serum at 198 ± 103.2 pg/ml. MSE and MIC-1 reduced IL-6 serum levels by 99% and 97% to 29 ± 19.8 pg/ml and 67 ± 55.2 pg/ml respectively with dexamethasone reducing the content by 98% at 46 ± 29.7 pg/ml (Fig. 5A)

MCP-1 showed no statistically significant differences among the treatment groups (Fig. 5. B), while TPA-induced KC serum levels were significantly reduced by MSE and MIC-1 by 63% and 59%, from 289 ± 196.8 pg/ml to 107 ± 21.14 pg/ml and 119 ± 41.1 pg/ml, respectively (Fig. 5C). Dexamethasone did not significantly reduce TPA-induced KC levels.

### MSE reduces the thickness in the ear tissue

**Fig. 6.**
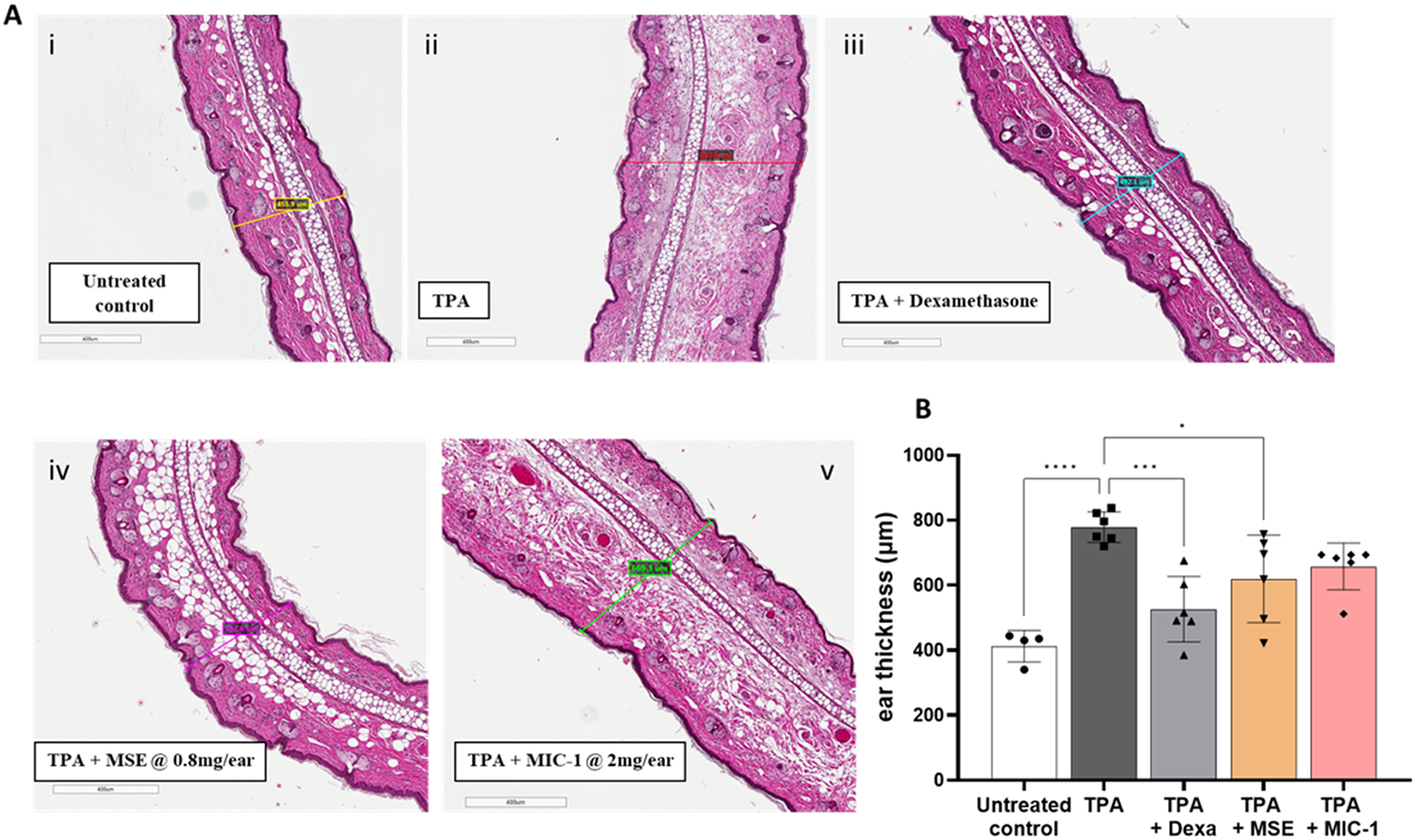
Thickness of transversal cuts of H&E-stained ears. Ear samples were collected at 4 h post-TPA administration. Treatment groups included ears that were left untreated (Untreated control) (i), ears treated with TPA (ii), TPA 20 min post-dexamethasone at 0.5 mg/ear (Dexa) (iii), MSE (2 mg/ear) (iv) or MIC-1 (0.8 mg/ear) (v). Images of H&E slides (**A**) were analyzed using Aperio ImageScope software. Data (**B**) is represented as mean ± SD of 4–6 ear samples. Statistical significance was determined by one-way ANOVA followed by Tukey’s post-hoc test; *p < 0.05, **p < 0.005, ***p < 0.0005, ****p < 0.0001.

Treatment-induced microscopic changes in ear morphology were evaluated using H&E staining on the ear biopsy samples. The Aperio ImageScope software was used to analyze the thickness of the H&E-stained slides at similar locations in the representative images of each of the treatments (Fig. 6). When comparing the average thickness of the cross section of the vehicle control ear samples, 412 ± 48.5 μm, there was a 47% thickness increase in the TPA-treated samples of 778 ± 47.1 μm, indicative of inflammation. MSE and MIC-1 increased the thickness of the TPA-treated ear samples by 33% and 38% to 618 ± 134.5 μm and 658 ± 72.2 μm, respectively. The dexamethasone-treated samples had a 21% increase to 526 ± 100.7 μm. MSE significantly (*p* < 0.05) attenuated the TPA-induced ear thickening. MIC-1 responded similarly to MSE, but the results were not statistically significant at *p* < 0.05.

## Discussion

This study reports the anti-inflammatory effect of an isothiocyanate-rich MSE and its main isothiocyanate, MIC-1, in a TPA-induced mouse ear edema model. Oral anti-inflammatory effects of MSE and MIC-1 were previously investigated in the *in vivo* sepsis model (Jaja-Chimedza et al., 2017), and ulcerative colitis model (Kim et al., 2017) which demonstrated that the specific anti-inflammatory benefits of MSE can be attributed to its high MIC-1 content. In addition, MSE was effective in the mouse metabolic syndrome model, where it improved insulin resistance and reduced body mass in mice fed high fat diet (Jaja-Chimedza et al., 2018). Both metabolic syndrome and diabetes are also associated with a low grade, systemic inflammation (Dandona et al., 2005).

This study demonstrates, for the first time, that topical application of MSE and MIC-1 inhibits inflammation in the TPA-induced mouse ear edema model (Figs. 2,3,6). The magnitude of the anti-inflammatory effects of MIC-1 and MSE containing 38% of MIC-1 were comparable to the positive control, dexamethasone, a glucocorticoid often used to treat inflammation-related skin diseases. The inhibition of skin inflammation after treatment with MSE or MIC-1 was associated with decreases in the pro-inflammatory cytokine and chemokines IL-6, MCP-1, and KC (Figs. 4,5). These mediator molecules play an essential role in the inflammation process. Lipidated peptidomimetics were shown earlier to also decrease cytokine and chemokine expression in the TPA-induced mouse ear inflammation model (Wu et al., 2020). Our study indicates that IL-6, MCP-1, and KC are critical players in the topical anti-inflammatory effect of MSE and MIC-1, likely mediated by the Nrf2 and NF-κB pathways (Sailaja et al., 2021).

In general, isothiocyanates, such as sulforaphane from Brassicaceae, are low yielding, unstable and liquid, while MIC-1 from MSE is a high yielding, stable white powder (Bertóti et al., 2019). Isothiocyanates from Brassicaceae have many documented health benefits such as anti-inflammatory, anticarcinogenic, antidiabetic, cardioprotective, antioxidant and antimicrobial (Yadav et al., 2022). MSE contains 38% MIC-1, because its precursor glucosinolate, glucomoringinin, is fully converted to MIC-1 under the MSE extraction method we developed (Jaja-Chimedza et al., 2017, Jaja-Chimedza et al., 2018). The primary mode of action of Brassicaceae isothiocyanates, i.e., sulforaphane, is the activation of Nrf2 signaling cascade (Nowicki et al., 2019). The results of this investigation further support our earlier observations that glycosylated moringa isothiocyanates, such as MIC-1, enhance Nrf2 signaling, at least as much as sulforaphane, but may also be active in the inhibition of the NF-κB signaling cascade (Sailaja et al., 2021). This work also supports the conclusion that MIC-1 may be the primary active component of MSE, since both produce similar pharmacological results at the same levels of MIC-1 (Waterman et al., 2015, Boyunegmez Tumer et al., 2015, Jaja-Chimedza et al., 2018, Jaja-Chimedza et al., 2017, Sailaja et al., 2022).

Skin inflammation is part of the defense against various environmental factors, pathogens, and chemical agents (Neagu et al., 2019). Keratinocytes, stimulated by these external causes, secrete pro-inflammatory cytokines that stimulate innate and adaptive immune responses (Awad et al., 2018). Secreted factors include molecules such as cytokines and growth factors, which affect multiple inflammatory processes (Sailaja et al., 2021, Bralley et al., 2008, García-Argáez et al., 2000, Liu et al., 2019a, Liu et al., 2019b, Park et al., 2001, Silvia et al., 2020). These are TNFα, the migration of T-helper (Th)1-polarized T lymphocytes, interleukins- 6,7,12,15,18, IFN-γ, or granulocyte-macrophage colony-stimulating factor (GM-CSF) as well as mediators that may reduce inflammation, such as IL-1Ra, IL-10, CXCL10 and prostaglandin E2 (PGE2) (Neagu et al., 2019). TPA, a phorbol ester, induces topical inflammation by activating protein kinase C, and a downstream transcriptional regulator of inflammation NF-кB (Neagu et al., 2019, Zhang et al., 2013, Hellweg et al., 2006). The TPA-induced ear edema is a robust and often used model to evaluate the anti-inflammatory effects of compounds (Sailaja et al., 2021, Bralley et al., 2008, García-Argáez et al., 2000, Liu et al., 2019a, Liu et al., 2019b, Park et al., 2001, Silvia et al., 2020). A single dose of TPA to the ears of mice could illicit ear swelling, thickness, redness, and infiltration of neutrophils and local and systemic secretion of cytokines and chemokines; all associated with an acute inflammatory response (Liu et al., 2019a, Park et al., 2001, Wu et al., 2020, Young et al., 1983).

This study demonstrated that both MSE and MIC-1 limit the expression of IL-6, MCP-1, and KC in the ear tissue (Fig. 4) and in the serum (Fig. 5) although the decrease in MCP-1in serum was not statistically significant at *p* < 0.05. Since very little to no IFN- γ, IL-1α, IL-1B, IL-10, IL-12p40, IL-12p70 and TNF-α were detected in the ear samples at the 4 h time point, 4 h may be too soon to detect the changes, or the changes were not detectable because of a low basal level of these inflammation mediators.

Various modes of action of the dexamethasone and other glucocorticoids include suppressing the migration of neutrophils and decreasing lymphocyte colony proliferation and the permeability of the capillary membranes. It also inhibits a variety of cytokines including IL-1, IL-12, IL-18, TNFα, IFN-γ and granulocyte-macrophage colony-stimulating factor (Noreen et al., 2021). It is tempting to speculate that combining MIC-1 with glucocorticoid drugs may have an additive, or even synergistic effect on reducing topical and systemic inflammation.

In conclusion, MIC-1 and MSE normalized for MIC-1 content effectively reduced skin inflammation in a TPA-induced ear edema model in a dose-dependent manner. Their effectiveness approached that of a widely used anti-inflammatory agent, dexamethasone. Upon treatment with both moringa seed-derived agents, there was an observable decrease in the TPA-induced ear edema, a decrease in the ear biopsy punch weights, a decrease in the dermal thickness, and a decrease in pro-inflammatory cytokines and chemokines MCP-1, KC, IL-6 and IL-1α in the ear tissue lysate. These results indicate that MSE and MIC-1 have the potential to mitigate skin inflammation and possibly treat diseases associated with skin inflammation. MIC-1-based remedies may be used alone or in combination with other anti-inflammatory agents.

## Abbreviations

MSE: moringa seed extract
MIC-1: moringa isothiocyanate-1
TPA: 12-O-tetradecanoylphorbol-13-acetate
NSAID: non-steroidal anti-inflammatory drug
CS: corticosteroids
ITCs: isothiocyanates
IL-1: interleukin-1
IL-6: interleukin-6
IL-12: interleukin-12
IL-18: interleukin-18
TNF-α: tumor necrosis factor-α
IFN-γ: interferon-gamma
NF-кB: nuclear factor-kappa B
MCP-1: monocyte chemoattractant protein-1
CXCL1(KC): CXC motif chemokine ligand 1
CXCR2: CXC motif chemokine receptor 2
AP-1: activator protein 1
NFATs: nuclear factor of activated T-cells
STATs: signal transducer and activator of transcription
Nrf2: nuclear factor erythroid 2-related factor 2
SAID: steroidal anti-inflammatory drug
IACUC: Institutional animal care and use committee
IL-1α: interleukin 1α
IL-1β: interleukin-1β
IL-10: interleukin-10
IL-12p40: interleukin-12p40
IL-12p70: interleukin-12p70
H&E: hematoxylin and eosin
